# By-Product of the Red Ginseng Manufacturing Process as Potential Material for Use as Cosmetics: Chemical Profiling and In Vitro Antioxidant and Whitening Activities

**DOI:** 10.3390/molecules27238202

**Published:** 2022-11-24

**Authors:** Hui-E Zhang, Meng-Yao Chu, Tao Jiang, Xin-Hong Song, Jian-Feng Hou, Li-Ye Cheng, Ye Feng, Chang-Bao Chen, En-Peng Wang

**Affiliations:** 1 Jilin Ginseng Academy, Changchun University of Chinese Medicine, Changchun 130117, China; 2 Shiqi Biological R&D Centre (Suzhou Industrial Park) Co., Ltd., Suzhou 215125, China; 3 Jilin Ji Test Technology Co., Ltd., Changchun 130117, China

**Keywords:** red ginseng, by-product, components, antioxidant and whitening activities

## Abstract

Red ginseng (RG), which is obtained from heated Panax ginseng and is produced by steaming followed by drying, is a valuable herb in Asian countries. Steamed ginseng dew (SGD) is a by-product produced in processing red ginseng. In the present study, phytochemical profiling of extracts of red ginseng and steamed ginseng dew was carried out using gas chromatography-mass spectrometry (GC-MS) and rapid resolution liquid chromatography coupled with quadrupole-time-of-flight mass spectrometry (RRLC-Q-TOF-MS) analysis. Additionally, antioxidant activities (DPPH, ·OH, and ABTS scavenging ability) and whitening activities (tyrosinase and elastase inhibitory activity) were analyzed. Phytochemical profiling revealed the presence of 66 and 28 compounds that were non-saponin components in chloroform extracts of red ginseng and steamed ginseng dew (RG-CE and SGD-CE), respectively. Meanwhile, there were 20 ginsenosides identified in n-butanol extracts of red ginseng and steamed ginseng dew (RG-NBE and SGD-NBE). By comparing the different polar extracts of red ginseng and steamed ginseng dew, it was found that the ethyl acetate extract of red ginseng (RG-EAE) had the best antioxidant capacity and whitening effect, the water extract of steamed ginseng dew (SGD-WE) had stronger antioxidant capacity, and the SGD-NBE and SGD-CE had a better whitening effect. This study shows that RG and SGD have tremendous potential to be used in the cosmetic industries.

## 1. Introduction

*Panax ginseng*, which belongs to the Araliaceae family, is a perennial herbaceous plant. It ranks first among the three most precious plantproducts from Northeast China, i.e., ginseng, marten, and Wula sedge [1]. The composition of ginseng is complicated, but the main components are ginseng saponins, volatile oil, various amino acids, minerals, and vitamins [2]. These ingredients have anti-cholesterol effects, promote subcutaneous capillary blood circulation, increase the skin’s nutritional supply, and prevent arteriosclerosis, which can delay skin aging [3]. In addition, the trace elements contained in ginseng can adjust the moisture balance of the skin, prevent dry skin, and increase skin elasticity [4]. Therefore, it can play a role in keeping the skin smooth and supple by preventing or reducing skin wrinkles. Since ancient times, ginseng has been known as “wrinkles to return to Dan.” Ginseng is an important medicinal material for antioxidants and anti-aging [5]. Red ginseng (RG) is produced from the root and rhizome of ginseng, a plant of the Araliaceae family, after it has been steamed and dried [6]. In China and South Korea, it is often used as a raw material for cosmetics. It is widely used in cosmetics and has whitening and anti-aging effects [7]. Steamed ginseng dew (SGD) is a by-product of the processing of red ginseng. Studies have found that SGD mainly contains a large number of semiterpenoids, sesquiterpenoids, a small amount of aliphatic and aromatic compounds, a small number of ginsenosides, and soluble polysaccharides. The main components are β-elemene, panaxydol, and panaxynol [8]. Pharmacological studies have shown that β-elemene has a strong inhibitory effect on cancer cell proliferation [9]. In addition, synergistic effects of various ingredients in ginseng oxygenol can destroy the permeability of the cell wall and cell membrane [10], which affects energy metabolism of bacterial substances and can inhibit the growth of some common bacteria, such as golden yellow grapes bacteria, picked bacteria, helicobacter pylori, etc [11]. Because of this antimicrobial role, it is widely used in various fields such as medicines and foods [12]. In the processing season in the main production area of Jilin Province, large amounts of SGD are abandoned or not fully utilized.

In recent years, the skin care value of red ginseng extract has received increasing attention and recognition. Studies have found that RG is rich in Rb1/Rg1/CK/Rg3 monomer saponins, which can inhibit the apoptosis of keratinocytes affected by ultraviolet radiation, promote light-induced DNA damage repair, and promote fibroblast synthesis [13]. Melanin is a natural and safe biopolymer that is synthesized in the melanosome of melanocytes [14]. However, excessive accumulation and overproduction of melanin can result in the development of physiological abnormalities such as pigment spots, chloasma, freckles, age spots, and even melanoma [15]. Melanogenesis is regulated by melano-genic enzymes, including tyrosinase, tyrosinase-related protein 1 and tyrosinase-related protein 2 [16]. Melanin synthesis inhibitors, including kojic acid and its derivatives, are tyrosinase inhibitors [17]. The cosmetic industry is developing products that contain various ingredients with skin whitening effects, such as kojic acid, to improve pigmentation [18]. Researchers have also found that Rg5 and Rk1 in RG can activate MEK-ERK signaling pathways, thus inhibiting the levels of tyrosine melanin activity [19]. In addition, RG displayed antioxidant activity in mice. Experiments found that red ginseng polysaccharides (RGPS) significantly increased the levels of glutathione peroxidase (GSH-Px) and SOD and decreased the content of the free radical oxidation product MDA in serum and tissues to reduce the damage to the body [20]. There are also related studies showing that RG can accelerate burn wound healing in mice [21]. The above literature shows that RG has antioxidant, whitening, and anti-aging pharmacological activities to a certain extent [22,23,24].

Although red ginseng has high medicinal value, it is expensive. To screen out a high-production and low-cost red ginseng substitute, this study analyzed the antioxidant activities, whitening activities, and chemical components of RG and SGD through vitro experiments combined with RRLC-Q-TOF-MS and GS-MS methods. First, the anti-aging and whitening effects of RG and SGD were evaluated by antioxidant activities, tyrosinase, and elastase inhibition experiments. Second, saponin constituents of RG and SGD were identified through RRLC-Q-TOF-MS. Finally, the chemical characteristics of RG-CE and SGD-CE were determined through GS-MS.

## 2. Results

### 2.1. Yield in Various Solvent Extracts

We investigated the yield of SGD extraction in different solvents. The yield of the chloroform, ethyl acetate, n-butanol, and aqueous extract ranged from 0.15 ± 0.022% to 86.28 ± 3.96% (Table 1). The high yield for the aqueous extract may have been due to the high content of water-soluble components (e.g., polysaccharides, salts, and proteins) in SGD extract.

### 2.2. Antioxidant Activity In Vitro

**Figure 1:**
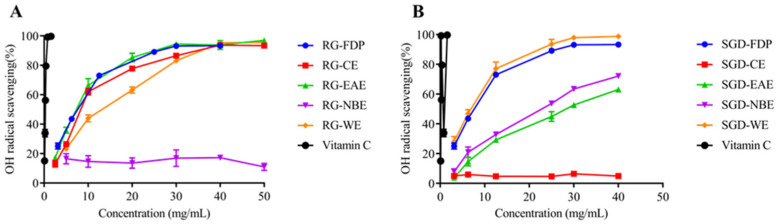
Antioxidant activity of RG and SGD extracts. (**A**) Hydroxyl radical scavenging activity of RG extracts. (**B**) Hydroxyl radical scavenging activity of SGD extracts. RG-FDP, freeze-dried powder of red ginseng; RG-CE, chloroform extract of red ginseng; RG-EAE, ethyl acetate extract of red ginseng; RG-NBE, n-butanol extract of red ginseng; RG-WE, water extract of red ginseng; SGD-FDP, freeze-dried powder of steamed ginseng dew; SGD-CE, chloroform extract of steamed ginseng dew; SGD-EAE, ethyl acetate extract of steamed ginseng dew; SGD-NBE, n-butanol extract of steamed ginseng dew; SGD-WE, water extract of steamed ginseng dew; vitamin C, positive control. Different groups indicate significant difference according to ANOVA followed by Tukey’s T-test(*p* < 0.05).

**Figure 2:**
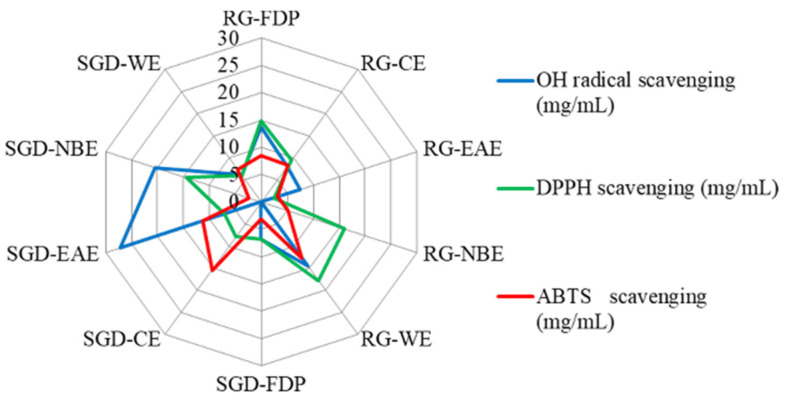
Comparative analysis of three antioxidant activities of RG and SGD extracts by radar chart. RG-FDP, freeze-dried powder of red ginseng; RG-CE, chloroform extract of red ginseng; RG-EAE, ethyl acetate extract of red ginseng; RG-NBE, n-butanol extract of red ginseng; RG-WE, water extract of red ginseng; SGD-FDP, freeze-dried powder of steamed ginseng dew; SGD-CE, chloroform extract of steamed ginseng dew; SGD-EAE, ethyl acetate extract of steamed ginseng dew; SGD-NBE, n-butanol extract of steamed ginseng dew; SGD-WE, water extract of steamed ginseng dew. (Blue symbols indicate hydroxyl radical scavenging activity, green symbols indicate DPPH free radical scavenging ability, and red symbols indicate ABTS radical scavenging activity. *n* = 3).

The free radical scavenging capacity (RSC) was evaluated according to the IC_50_ value and scavenging percentage (IC_50_ value is defined as the concentration of the antioxidant needed to scavenge 50% of free radicals present in the test solution). The smaller the IC_50_ value, the stronger the antioxidant activity [25]. It can be seen that the scavenging capacity of ·OH by different RG extract fractions was in the order of EAE > CE > WE > FDP, and EAE had a better scavenging effect on ·OH, with IC_50_ values of 7.25 ± 0.54 mg/mL and 7.46 ± 0.32 mg/mL, respectively. The order of ·OH scavenging ability of SGD fractions was WE > FDP > NBE > EAE, and the IC_50_ values were 6.09 ± 0.27 mg/mL, 6.82 ± 0.23 mg/mL, 20.43 ± 0.14 mg/mL, and 27.24 ± 0.56 mg/mL, respectively. The ability of radical scavenging activity is similar in EAE and NBE of SGD (Figure 1 and Figure 2).

**Figure 3:**
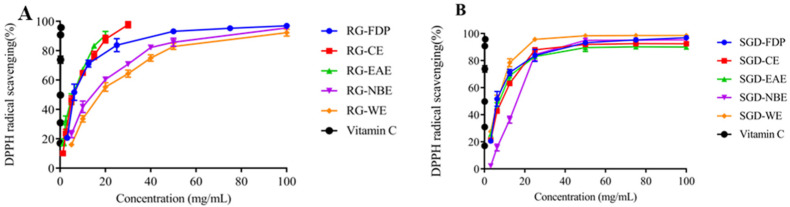
Antioxidant activity of RG and SGD extracts. (**A**) DPPH free radical scavenging ability of RG and its extracts. (**B**) DPPH free radical scavenging ability of SGD extracts and its extracts. RG-FDP, freeze-dried powder of red ginseng; RG-CE, chloroform extract of red ginseng; RG-EAE, ethyl acetate extract of red ginseng; RG-NBE, n-butanol extract of red ginseng; RG-WE, water extract of red ginseng; SGD-FDP, freeze-dried powder of steamed ginseng dew; SGD-CE, chloroform extract of steamed ginseng dew; SGD-EAE, ethyl acetate extract of steamed ginseng dew; SGD-NBE, n-butanol extract of steamed ginseng dew; SGD-WE, water extract of steamed ginseng dew; vitamin C, positive control. Different groups indicate significant difference according to ANOVA followed by Tukey’s T-test (*p* < 0.05).

The DPPH radical scavenging capacity of various solvent extracts of RG was ordered as EAE > CE > FDP > WE > NBE. The DPPH radical scavenging capacity of EAE was greater. Accordingly, its IC_50_ values were 3.4 ± 0.20 mg/mL and 2.45 ± 0.69 mg/mL, respectively. The DPPH free radical scavenging capacity of each polar extract of SGD was ordered as WE > FDP > EAE > CE > NBE, and the IC_50_ values were 5.95 mg/mL, 6.76 ± 0.31 mg/mL, 7.10 ± 0.24 mg/mL, 7.90 ± 0.25 mg/mL, and 14.45 ± 0.32 mg/mL, respectively (Figure 2 and Figure 3). When the sample concentration was 0–20 mg/mL, the DPPH radical scavenging rate increased with the increase in sample concentration. The scavenging percentage tended to be mild when the sample concentration was more than 20 mg/mL. (Figure 1, Figure 2 and Figure 3).

**Figure 4:**
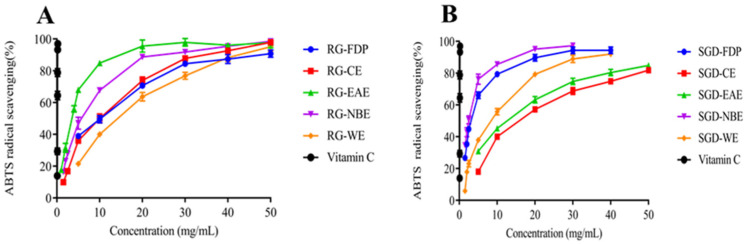
Antioxidant activity of RG and SGD extracts. (**A**) ABTS radical scavenging activity of RG extracts. (**B**) ABTS radical scavenging activity of SGD extracts. RG-FDP, freeze-dried powder of red ginseng; RG-CE, chloroform extract of red ginseng; RG-EAE, ethyl acetate extract of red ginseng; RG-NBE, n-butanol extract of red ginseng; RG-WE, water extract of red ginseng; SGD-FDP, freeze-dried powder of steamed ginseng dew; SGD-CE, chloroform extract of steamed ginseng dew; SGD-EAE, ethyl acetate extract of steamed ginseng dew; SGD-NBE, n-butanol extract of steamed ginseng dew; SGD-WE, water extract of steamed ginseng dew; vitamin C, positive control. Different groups indicate significant difference according to ANOVA followed by Tukey’s T-test (*p* < 0.05).

The ABTS scavenging assay was used to measure the antioxidant capacity of the various solvent extracts of RG and SGD as presented in Figure 1, Figure 2, Figure 3 and Figure 4. The EAE of RG had a stronger scavenging effect. The IC_50_ values were 2.93 ± 0.04 mg/mL and 3.19 ± 0.08 mg/mL, respectively. The NBE scavenging ability of RG was superior. The IC_50_ value was 5.19 ± 0.17 mg/mL. The ABTS^+^ radical scavenging abilities of various solvent extracts of SGD were different from those of RG. SGD-NBE, which had an IC_50_ of 2.40 ± 0.03 mg/mL, had a higher ABTS radical scavenging ability than SGD-FDP, which had an IC_50_ of 3.14 ± 0.11 mg/mL.

### 2.3. Inhibitory Activities against Tyrosinase and Elastase

**Figure 5:**
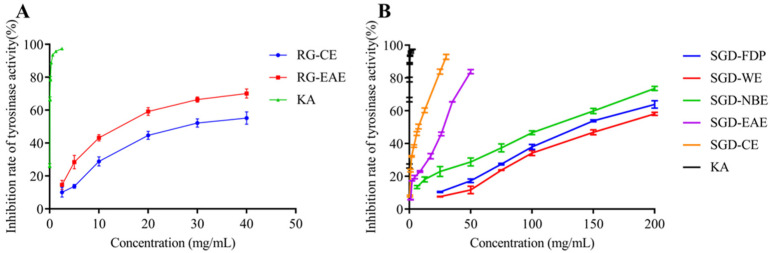
Inhibitory activity of samples on tyrosinase. (**A**) Tyrosine inhibition rate of RG extracts. (**B**) Tyrosine inhibition rate of SGD extracts. RG-CE, chloroform extract of red ginseng; RG-EAE, ethyl acetate extract of red ginseng; KA, kojic acid (positive control); SGD-FDP, freeze-dried powder of steamed ginseng dew; SGD-CE, chloroform extract of steamed ginseng dew; SGD-EAE, ethyl acetate extract of steamed ginseng dew; SGD-NBE, n-butanol extract of steamed ginseng dew; SGD-WE, water extract of steamed ginseng dew.

**Figure 6:**
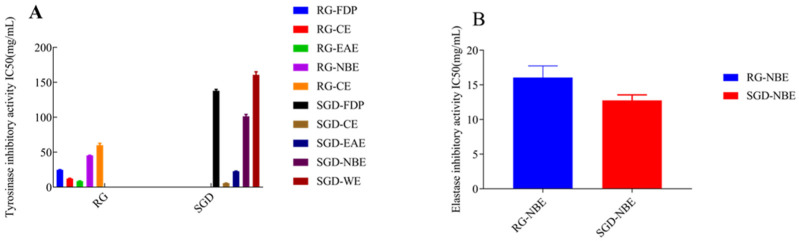
Changes in IC_50_ values on tyrosinase and elastase activities. (**A**) The IC_50_ values of RG and SGD extract on tyrosinase activity. (**B**) The IC_50_ values of RG-NBE and SGD-NBE on elastase activity. RG-CE, chloroform extract of red ginseng; RG-NBE, n-butanol extract of red ginseng; RG-EAE, ethyl acetate extract of red ginseng; SGD-FDP, freeze-dried powder of steamed ginseng dew; SGD-CE, chloroform extract of steamed ginseng dew; SGD-EAE, ethyl acetate extract of steamed ginseng dew; SGD-NBE, n-butanol extract of steamed ginseng dew; SGD-WE, water extract of steamed ginseng dew.

By tyrosinase-catalyzed oxidation, L-tyrosine is converted to the red-brown dopaquinone via l-3, 4-dihydroxy phenylalanine; dopaquinone is further oxidized to yield the mostly brown to black colored polymeric melanins, which are responsible for blackened skin. Tyrosinase, which has a binuclear copper cluster, in the common mushroom (Agaricus bisporus) and human malignant melanoma, is a crucial enzyme in melanogenesis [26]. The tyrosinase inhibitory activities of RG and SGD are shown in Figure 5 and Figure 6. The tyrosinase-inhibitory activity (IC_50_) of SGD-CE was 5.76 ± 0.10 mg/mL, which is more potent than RG-EAE (8.64 ± 0.36 mg/mL) (*p* < 0.05). Elastase activity affects collagen content. The elastic fiber network plays an important role in sustaining skin elasticity, and the decrease of skin elasticity is an essential factor in the formation of wrinkles [27]. The elastase inhibitory activities (IC_50_) of RG and SGD are shown in Figure 6, and SGD-NBE had a higher activity (12.75 ± 0.81 mg/mL) than RG-NBE (16.06 ± 1.68 mg/mL) (*p* < 0.05).

### 2.4. Analysis of Tyrosinase Inhibition

To confirm the reversibility of RG- and SGD-mediated inhibition, plots of enzyme activity were constructed (Supplementary Materials Figure S1). The results showed straight lines passing through the origin, indicating RG- and SGD-mediated reversible inhibition. Lineweaver–Burk plot analysis was performed to assess tyrosinase inhibition by RG and SGD. We adopted the Lineweaver–Burk plot analysis to elucidate the inhibition type and mechanism of RG and SGD on tyrosinase. The results showed changes in both the appearance of the Vmax and Km, indicating that RG-EAE, SGD-FDP, and SGD-WE induced a mixed type of inhibition. Moreover, SGD-EAE and SGD-NBE induced anticompetitive inhibition, and SGD-CE induced competitive inhibition (Supplementary Materials Figure S2).

### 2.5. Analysis of Elastase Inhibition

To understand the inhibitory mode of RG and SGD against elastase, a kinetic study was performed based on the IC_50_ calculation to evaluate the inhibition type and calculate the inhibition constant. The kinetics results of the enzyme from the Lineweaver–Burk plot of 1/V versus substrate N-succinyl-Ala-Ala-Ala-pnitroanilide 1/[S] in the presence of different inhibitor concentrations showed a series of straight lines (Supplementary Materials Figure S3). The result of the Lineweaver–Burk plot showed that Vmax remains the same without significantly affecting the slopes. Km increased with increasing concentration, while Vmax remained the same with an insignificant difference. This behavior indicated that SGD-NBE competitively inhibited the enzyme (Supplementary Materials Figure S4) and RG-NBE belonged to mixed inhibition.

### 2.6. Identification of Ginsenosides

The RRLC-Q-TOF-MS method was used to analyze the saponin components of the RG-NBE and SGD-NBE fractions with good whitening and anti-aging activities in ESI^-^ mode (Table 1). In the first-order mass spectra of ginsenosides in ESI^-^ mode, quasi-molecular ions mainly exist in the form of [M−H]^−^ and [M+HCOOH]^−^ [28]. After the molecular composition was determined by the detected molecular ion–isotope abundance ratio, the tandem mass spectrometry results were further compared with the self-built database and literature for analytical calibration. The accurate molecular weight, tandem mass spectrometry data, and retention time of the two were compared, and the saponins were identified [29].

**Figure 7:**
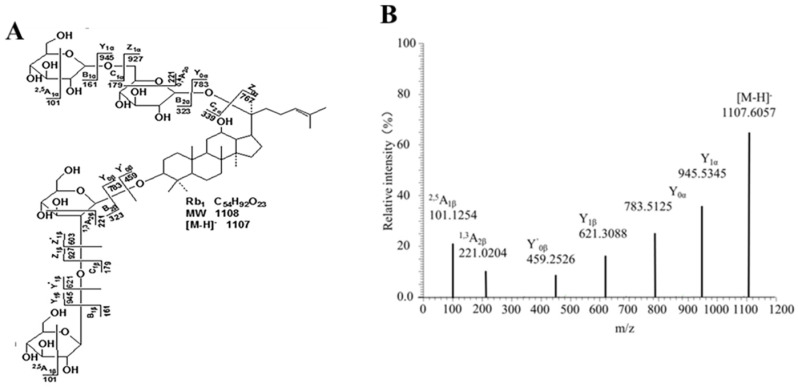
Structure and secondary mass spectrum of Rb1. (**A**) Structure diagram. (**B**) Secondary mass spectrometry.

Taking protopanaxadiol ginsenoside Rb1 as an example, the fragmentation mode and secondary spectrum are shown in Figure 7. [M−H]^−^ (*m*/*z* 1107.6057) was the quasi-molecular ion peak, and under certain energy collisions, [M−H]^−^ will be further broken. [M−H]^−^ fragmentation involving loss of a molecular glucose residue produces Y1α (*m*/*z* 945) ion fragments [30,31]. [M−H]^−^ Fragmentation involving loss of two molecular glucose residues produces Y0α (*m*/*z* 783) ion fragments. Y′1β (*m*/*z* 621) ion fragments were generated when two glucose residues were lost on the α-chain and one glucose residue was lost on the β chain. Y′0β (*m*/*z* 459) ion fragments were produced by the simultaneous loss of two glucose residues in the α and β chains. Cross-loop cleavage of sugar chains will produce 2,5A1α/2,5A1β (*m*/*z* 101) ion fragments and 0,4A2α/1,3A2β (*m*/*z* 221) ion fragments.

The chemical components of the NBE of RG and SGD were mostly ginsenosides (Table 2). Due to the short time of high-temperature steam in the processing, some rare ginsenosides such as Rg3, Rg2, Rg5, Rg6, Rk1, and F4, were not detected with low content, but there were some natural malonyl ginsenosides such as mRd, mRc, mRb2, and mRb3. Most of the malonyl ginsenosides will be hydrolyzed to remove the malonyl and produce the corresponding ginsenosides after a long time of high-temperature processing [32]. For example, mRb1 was hydrolyzed to Rb1, so the content of ginsenosides in processed products similar to Rb1 was significantly increased. Rb1 and Rg1 were detected in all NBEs. 20 ginsenosides were identified by RRLC-Q-TOF-MS from NBE of RG and SGD. There were many kinds of rare saponins in the NBE of RG and SGD, such as Rg3, Rg2, Rg5, Rg6, Rk1, and F4.

### 2.7. Identification of RG-CE and SGD-CE with GC-MS

Table 3 compiles all identified compounds from the GC-MS measurements of SGD-CE. The data were retrieved by the National Bureau of Standards and Technology (NIST) 2011 standard mass spectrometry library, and the chromatographic peaks with matching degrees higher than 80 were selected. Combined with the relevant literature, the chromatogram was analyzed to determine the chemical composition, and the relative percentage content of each component was calculated by the peak area normalization method.

**Figure 8:**
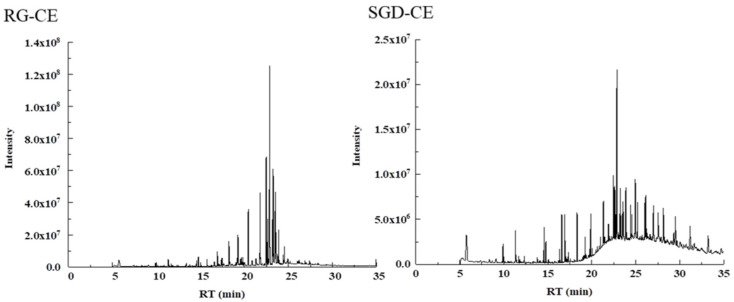
TIC of RG-CE and SGD-CE with GC-MS.

**Figure 9:**
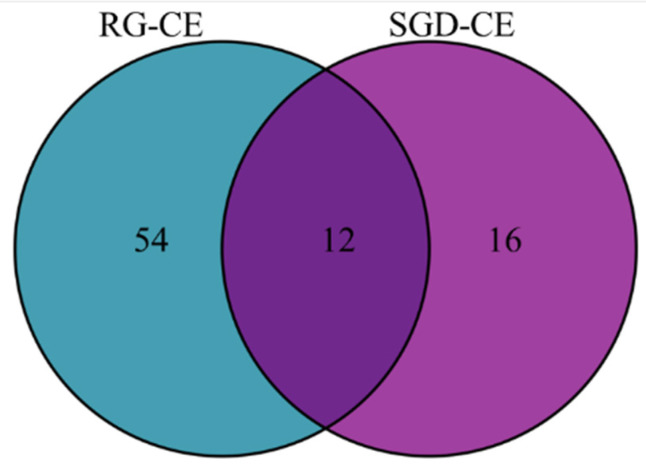
Venn diagram of similarities and differences of volatile compounds in RG-CE and SGD-CE.

228 soluble constituents of RG-CE were isolated by GC-MS, and 66 compounds were identified therein; the results are shown in Table 3 and Figure 8. Additionally, 203 soluble constituents were isolated from SGD-CE, and 28 compounds were identified. The results are shown in Table 3 and Figure 8. According to Figure 9, the 12 common compounds in RG-CE and SGD-CE, most of which were non-saponins, were analyzed using a Venn diagram. In recent years, many pharmacological effects of non-saponin components have been reported. Related studies have found that panaxydol and panaxytriol have the effect of lipid peroxidation inhibition [33]. Previous studies have identified hexadecanoic acid, octadecanoic acid, oleic acid, β-sitosterol, linoleic acid, and linolenic acid as important active substances for antioxidant and tyrosinase inhibitory effects as well [34,35]. In this study, by comparing the volatile components in SGD with those of RG reported in the literature, it was found that they also have similar active substances.

## 3. Discussion

In this study, the chemical constituents, antioxidant activities, and whitening activities of RG and SGD were compared. It can be seen from Table 2 that there are 20 common saponin components in the RG-NBE and SGD-NBE, which mainly consist of Rg1, Re, Rg3, Rb1, etc. Recent reports have shown that ginsenosides modify skin physiology and mitigate skin disorders such as photo-ageing and hyperpigmentation. In addition, protopanaxatriol dramatically suppressed the expression of genes encoding the melanogenic proteins tyrosinase and tyrosinase-related protein-1 and -2 [36]. Ginsenoside Rg3 can activate ERK in melanocytes, reduce intracellular tyrosinase activity, and ultimately inhibit melanin production [37]. Ginsenoside Rb2 not only has antioxidant effects, but it also inhibits tyrosinase activity and reduces melanin production [38]. From the comparative analysis of the volatile components of the RG-CE and SGD-CE depicted in Table 3, it was found that there were 12 volatile components in both. The highest content of panaxydol in the RG-CE was 11.258%, while the highest content of phenol,2,2′-methylenebis[6-(1,1-di-methylethyl)-4-methyl in the SGD-CE was 14.025%. Both extracts contained a high content of phenol,2,2′-methylenebis[6-(1,1–dimethyleth yl)-4-methyl, which was especially evident in the SGD-CE, which was up to 14.025%. The contents of 1,9-diphenyl-1,3,5,7-nonatetraene and phenol, 2,2′-methylenebis[6-(1,1-dimethylethy l)-4-methyl] in two kinds of chloroform extracts were more than 5.00%.

RG and SGD are derived from the same plant. Antioxidant and whitening activity results showed that SGD, as a by-product of RG, has similar antioxidant and whitening effects as RG and has great potential in the development of food and cosmetics based on SGD. SGD is rich in resources and is often treated as industrial waste. To reuse waste resources, it was found that there are also great differences in chemical composition. In terms of the distribution of components, RG was significantly higher than SGD. The 82 components identified, which only contain 12 common components, are mainly alkane compounds, such as nonane, 4,5-dimethyl-, nonane, 3-methyl-5-propyl, etc. There are also large differences in the content of these components. In addition, the RG-CE contained amides, alcohols, phenols, and alkenes, such as 2,4-dioxohexahydro1,3,5-triazine, panaxydol, phenol,2,2′-methylenebis[6-(1,1-dimethylethyl)-4-methyl, and squalene, while the SGD-CE included more alkanes and phenolic compounds, such as heptadecane, heneicosane, nonacosane, phenol, 2,2′-methylenebis[6-(1,1-dimethylethyl)-4-methyl, etc. Through literature review, it was found that the volatile components in the RG-CE and SGD-CE included a variety of effective active components. For example, panaxydol is one of the components of alkynols in the Panax ginseng C.A. Meyer (Araliaceae family), which significantly prevents tissue degradation in rats and increases antioxidant levels [39]. Studies have shown that the antioxidant capacity of squalene is stronger than other lipid molecules in the skin [40]. Squalene in the skin effectively inhibits lipid peroxidation, which in turn helps the skin resist damage caused by UV radiation and other oxidative reactions [41]. Squalene also has moisturizing effects [42]. RG and SGD contain more common active ingredients, suggesting that there may be some correlation between the differences between RG and SGD on ingredients and their efficacy. However, studies on the inhibition of tyrosinase activity by volatile components of RG and SGD have not been reported.

## 4. Materials and Methods

### 4.1. Materials

Red ginseng and SGD freeze-dried powder was used in this experiment. Steamed ginseng dew (SGD) and 5-year-old RG were purchased from Fusong County agricultural planting development Co (Baishan, Jilin, China).

### 4.2. Sample Preparation

Different polar extracts were prepared by the method in reference [43] with slight adjustments. SGD was filtered to remove impurities and placed in a rotary evaporator at 60 °C. The concentrated liquid was transferred to a freeze dryer to obtain steamed ginseng dew freeze-dried powder (SGD-FDP). Similarly, a certain amount of red ginseng powder was extracted 20 times with different concentrations of ethanol (95%, 75%, 55%) and distilled water. The extract was combined and placed in a rotary evaporator at 60 °C to decompress and concentrate. The concentrated liquid was transferred to the freeze dryer to obtain the red ginseng freeze-dried powder (RG-FDP). A certain quantity of freeze-dried powder of each sample was then accurately obtained and dissolved in distilled water. Meanwhile, the petroleum ether, ethyl acetate, chloroform, and water-saturated n-butanol were extracted at a ratio of 1:1, and each was extracted three times. The extraction solution was combined and concentrated under reduced pressure at 60 °C in a water bath to obtain the petroleum ether phase, ethyl acetate phase, chloroform phase, water-saturated n-butanol phase, and water phase. The extraction rate was weighed and calculated.

### 4.3. Evaluation of In Vitro Antioxidant Activity

#### 4.3.1. Hydroxyl Radical Assay

The hydroxyl radical scavenging activity was assayed according to references [44,45]. The EAE and CE of RG and SGD were dissolved in 20% DMSO, and the other extracts were dissolved in distilled water. Briefly, RG and SGD extract solutions, 50 µL of an aqueous solution of FeSO_4_ (FeSO_4_·7H_2_O) (3 mM) and 50 µL of an aqueous solution of H_2_O_2_ (3 mM), were mixed in a microplate and incubated for 10 min. Subsequently, 50 µL of an aqueous solution of salicylic acid (6 mM) was added and incubated at room temperature for 30 min in the dark. The absorbance of the sample was recorded at 510 nm. The IC_50_ values were calculated and expressed as the mean ± SD in mg/mL.

#### 4.3.2. DPPH Scavenging Activity Assay

The DPPH scavenging activity was assayed according to reference [46]. Briefly, RG and SGD extract solutions and 100 µL of DPPH in methanol (50 µM) were mixed in a microplate plate and allowed to stand at room temperature for 30 min in the dark. The absorbance of the sample was recorded at 517 nm. The half-maximal inhibitory concentration (IC_50_) values (the concentrations required to scavenge 50% of the DPPH radicals present in the test solution) were calculated and expressed as the mean ± standard deviation (SD) in mg/mL.

#### 4.3.3. ABTS^+^ Scavenging Activity Assay

The ABTS^+^ scavenging activity was assayed according to our previous method [47]. Briefly, 80 μL of diluted ABTS^+^ solution and 20 μL of sample solutions were mixed in a microplate and incubated for 20 min in the dark. The absorbance of the sample was recorded at 734 nm. The IC_50_ values were calculated and expressed as the mean ± SD in mg/mL.

### 4.4. Kinetics Assay

#### 4.4.1. Tyrosinase and Elastase Inhibition

L-Tyrosine oxidation by tyrosinase was spectrophotometrically determined according to reference [48]. L-DOPA and PBS buffer solutions were mixed by shaking and reacted at room temperature for 10 min in the dark. The sample and tyrosinase solution (800 U/mL) were added in turn and mixed by shaking and incubated at 37 °C for 5 min. The absorbance of the sample was recorded at 475 nm. The positive control was kojic acid. The IC_50_ values from the graph plots of the dose–response curves at each sample concentration generated using Graphpad Prism software 7.0 (San Diego, CA, USA) were calculated and expressed as the mean ± SD in mg/mL. The inhibition of elastase activity was evaluated spectrophotometrically by the method of Park et al. [49,50,51].

#### 4.4.2. Kinetic Analysis for the Tyrosinase Inhibition

According to reference [52], to begin the assay, a 50-μL sample of enzyme solution was added to 1 mL of the reaction mix. Enzyme activity was recorded as the change in absorbance at 475 nm using a spectrophotometric tyrosinase. To describe the inhibition type using the reciprocal (1/[S]) of the concentration of the substrate L-DOPA as the *X*-axis and the reciprocal of the reaction rate (1/V) as the *Y*-axis to draw a Lineweaver–Burk double-reciprocal curve to calculate the kinetic parameters of aminoacidase to determine the type of reversible inhibitory activity of RG and SGD on tyrosinase.

#### 4.4.3. Kinetic Analysis for Elastase Inhibition

Kinetic analysis was carried out to determine the mode of inhibition [53]. The inhibition type was determined based on the IC_50_ value. Kinetics was carried out by varying the concentration of N-succinyl-Ala-Ala-Ala-p-nitroanilide in the presence of different concentrations of RG-NBE and SGD-NBE. Briefly, the N-succinyl-Ala-Ala-Ala- p-nitroanilide concentration was changed between 0, 0.25, 0.5, 1, 2, and 3 mg/mL for its kinetics studies, and the remaining procedure was the same for all kinetic studies as described in an elastase inhibition assay. Maximal initial velocities were determined from the initial linear portion of absorbances up to 10 min after the addition of enzyme at per-minute intervals. The inhibition type on the enzyme was assayed by the Lineweaver–Burk plot of inverse velocities (1/V) versus the inverse of substrate concentration 1/[S].

### 4.5. Mass Spectrographic Assay

#### 4.5.1. Liquid Chromatographic and Mass Spectrometric Conditions

RRLC-Q-TOF-MS analyses were performed to detect and compare the ginsenoside contents of RG-NBE and SGD-NBE. The sample injections were separated by liquid chromatography using a ZORBAX SB-C18 column (3.0 mm × 5.0 cm, 2.7 μm) at 30 °C, with 0.1% formic acid (*v*/*v*) and acetonitrile used as mobile phases A and B, respectively. The gradient elution began with 15% B and then was programmed as follows: 19% from 0 min to 5 min, 19% from 5 min to 10 min, 25% from 10 min to 13 min, 28% from 13 min to 15 min, 28% from 15 min to 18 min, 30% from 18 min to 22 min, 35% from 22 min to 25 min, 40% from 25 min to 30 min, 60% from 30 min to 35 min, and 100% from 35 min to 40 min. The flow rate was 0.3 mL/min, and the injected sample volume was 5 μL.

The mass spectrometer was operated in negative ion mode. The optimized mass spectrometry conditions were as follows: nebulizer at 30 psig, the capillary voltage of 3500 V, cone voltage of 65 V, fragmentation voltage of 220 V, drying gas temperature of 350 °C, drying gas (N_2_) flow rate of 8 L/min, atomization gas pressure of 2.41 × 10^5^ Pa, and a mass-scanning range of *m*/*z* 100~2000. Data analysis was performed using Agilent Mass Hunter (B.03.01).

#### 4.5.2. GC-MS Measurements for Volatile Identification

GC-instrument (Thermo Fisher TRACE 1310 GC-Triple Quadrupole MS) using electron ionization (EI) was used for SGD-CE measurements for the identification of volatile organic compounds (VOCs). The column used was 30 mm long and had an inner diameter of 0.25 mm with a 0.25 µm thickness (HP-5 ms). Separation was achieved by the following temperature program: initial 50 °C with a 1 min hold, ramped at 8 °C/min to 120 °C (5 min hold) and then ramped at 5 °C/min to 290 °C. A splitless injection port was held at 240 °C. The splitless injection was used, with a splitless time of 1.0 to 3.0 min from the injection. Helium (≥99.99%) was used as a carrier gas at a constant flow rate of 1.0 mL/min. The MS operation parameters were as follows: mass scan range of 50–550 *m*/*z*, ion source temperature of 230 °C, ionization energy of 70 eV, and GC-MS transfer line temperature of 280 °C.

Results were assessed using Agilent’s GC-MS solution workstation, NIST 11 American standard database, combined with reference substance comparison, compound mass spectrometry fragmentation rules, and reference to the reported literature. The relative content of the compounds was calculated by the peak area normalization method.

### 4.6. Statistical Analysis

All experiments were carried out in triplicate. The results in Table 1 and Figure 1, Figure 2, Figure 3, Figure 4 and Figure 5 are expressed as mean ± standard deviation (S.D.). Data obtained were analyzed by one-way analysis of variance (ANOVA) followed by Tukey’s post hoc tests using IBM SPSS Statistics 21.0. (SPSS Inc., Chicago, IL, USA). In Table 2 and Table 3, the differences of each compound in the RG and SGD were compared. In Figure 6, IC_50_ of tyrosinase and elastase activities on RG extracts and SGD extracts were compared. A *p*-value less than 0.05 was used to indicate a statistically significant result.

## 5. Conclusions

The present work aimed to investigate the chemical composition of RG and SGD and to explore their antioxidant and whitening activities in the quest to find ingredients to be used in cosmetic formulations. Results regarding antioxidant and whitening activities showed that RG and SGD had similar anti-aging and whitening effects. The LC-MS analysis revealed that their major constituents were saponins. GC-MS analysis showed that there are 12 volatile components in RG and SGD. Therein, the relative content of 2,4-Di-tertbutylphenol and phenol, 2,2′-methylene bis [6-(1,1, dimethylethyl)-4-methyl is higher, which may also be the main factor controlling the antioxidant activity of SGD. In addition, SGD has the characteristics of large production and low price. To make full use of ginseng resources, SGD has great application potential in cosmetics development.

## Figures and Tables

**Table 1:** Extracting rate of RG and SGD in each polar part (%, *n* = 3).

Samples	Freeze-Dried Powder (FDP)	Chloroform Extract (CE)	Ethyl Acetate Extract (EAE)	N-Butanol Extract (NBE)	Water Extract (WE)
RG	39.88 ± 2.59	1.46 ± 0.08	0.91 ± 0.11	25.93 ± 2.78	64.19 ± 3.82
SGD	1.40 ± 0.02	0.15 ± 0.02	0.57 ± 0.05	8.51 ± 0.83	86.28 ± 3.96

**Table 2:** The common peaks of constituents of RG-NBE and SGD-NBE (*n* = 3).

No.	Identity	Formula	MW	RG	SGD	[M−H]^−^	[M+COOH]^−^	MS/MS Fragment Ion (*m*/*z*)
1	Noto R_1_	C_47_H_80_O_18_	932.535	+	+	931.527	977.533	977[M+HCOO]^−^; 931[M−H]^−^; 799[M−H−Xyl]^−^;
								6375[M−H−Xyl−Glc]^−^; 4758[M−H−Xyl−GlcGlc]^−^;
								293[XylGlc−H_2_O−H]^−^; 179[Glc−H]^−^; 1615[Glc−H_2_O−H]^−^;
								_β_149[Xyl−H]^−^; 1311 [Xyl−H_2_O−H]^−^; ^1,5^A1_α/_^2,5^A_1β_101
2	Rg_1_	C_42_H_72_O_14_	800.492	+	+	799.485	845.490	799[M−H]^−^; 637[M−H−Glc]^−^; 475[M−H−GlcGlc]^−^;
								391[M−H−GlcGlc−C_6_H_12_]^−^; B_1α_/B_1β_161; ^2,5^A_1α_/^2,5^A_1β_101
3	Re	C_48_H_82_O_18_	946.550	+	+	945.543	991.548	945[M−H]^−^; 783[M−H−Glc]^−^; 637[M−H−Glc−Rha]^−^;
								475[M−H−GlcGlc−Rha]^−^
4	Rf	C_42_H_72_O_14_	800.492	+	+	799.485	845.490	799[M−H]^−^; 637[M−H−Glc]^−^; 475[M−H−GlcGlc]^−^;
								391M−H−GlcGlc−C_6_H_12_]^−^; ^1,3^A_2β_221; ^2,5^A_1β_101
5	Noto R_2_	C_41_H_70_O_13_	770.482	+	+	769.474	793.471	769[M−H]^−^; 637[M−H−Xyl]^−^; 475[M−H−Xyl−Glc]^−^;
								391[M−H−Xyl−Glc−C_6_H_12_]^−^
6	Ra_1_	C_58_H_98_O_26_	1210.635	+	+	1209.627	1255.633	1209 [M−H]^−^; 1077[M−H−Xly]^−^; 945[M−H−Xly−Ara(p)]^−^;
								783[M−H−Xly−Ara(p)−Glc]^−^; 323[GlcGlc−H]^−^;^2,4^A_2α_191
								621[M−H−Xly−Ara(p)−GlcGlc]^−^;
								459[M−H−Xly−Ara(p)−Glc GlcGlc]^−^;
7	Ra_2_	C_58_H_98_O_26_	1210.635	+	+	1209.627	1255.633	1209 [M−H]^−^; 1077[M−H−Xly]^−^; 945[M−H−Xly−Ara]^−^;
								783[M−H−Xly−Ara−Glc]^−^; 621[M−H−Xly−Ara−GlcGlc]^−^;
								459[M−H−Xly−Ara−GlcGlcGlc]^−^; 323[GlcGlc−H]^−^;
								^2,4^A_2α_191
8	Rb_1_	C_54_H_92_O_23_	1108.603	+	+	1107.596	1153.601	1107[M−H]^−^; 945[M−H−Glc]^−^; 783[M−H−GlcGlc]^−^;
								621[M−H−GlcGlcGlc]^−^; 459[M−H−GlcGlcGlcGlc]^−^;
								B_2α_/B_2β_323[GlcGlc−H]^−^; C_1α_/C_1β_179[Glc−H]^−^
9	Rc	C_53_H_90_O_22_	1078.592	+	+	1077.585	1123.591	1077[M−H]^−^; 945[M−H−Ara(f)]^−^; 783[M−H−Ara−Glc]^−^;
								621M−H−Ara−Glc Glc]^−^; 459[M−H−Ara−GlcGlcGlc]^−^
10	Rb_2_	C_53_H_90_O_22_	1078.592	+	+	1077.585	1123.591	1077[M−H]−; 945[M−H−Ara(p)]^−^; 783[M−H−Ara−Glc]^−^;
								621[M−H−Ara−GlcGlc]^−^; B_2α_293[AraGlc−H]^−^; ^0,4^A_2α_191;
								C_1α_149 [Ara−H]^−^; ^2,5^A_1β_101
11	Rb_3_	C_53_H_90_O_22_	1078.592	+	+	1077.585	1123.591	1077[M−H]^−^; 945[M−H−Xyl]^−^; 83[M−H−Xyl−Glc]^−^;
								621[M−H−Xyl−GlcGlc]^−^; B_2α_293[XylGlc−H]^−^;
								C1α149[Xyl−H]^−^
12	Rd	C_42_H_72_O_13_	784.497	+	+	945.543	991.548	945[M−H]^−^; 783[M−H−Glc]^−^; 621[M−H−GlcGlc]^−^;
								459[M−H−GlcGlcGlc]^−^; B_1α_/B_1β_161[Glc−H]^−^;
								^2,5^A_1α_/^2,5^A_1β_101
13	Rs_1_	C_55_H_92_O_23_	1120.603	+	+	1119.596	1165.601	1119[M−H]^−^; 1077[M−H−Ac]^−^; 945[M−H−Ac−Xly]^−^;
								783[M−H−Ac−Xly−Glc]^−^; 621[M−H−Ac−Xly−GlcGlc]^−^;
								459[M−H−Ac−Xly−GlcGlcGlc]^−^; 293[XylGlc−H]^−^;
								C_1α_149[Xyl−H]^−^
14	Rs_2_	C_55_H_92_O_23_	1120.603	+	+	1119.596	1165.601	1119[M−H]^−^; 1077[M−H−Ac]^−^;945[M−H−Ac−Ara(f)]^−^;
								783[M−H−Ac−Ara(f)−Glc]^−^; C_1α_149[Ara(f)−H]^−^;
								459[M−H−Ac−Ara(f)−GlcGlcGlc]^−^; 293[Ara(f)Glc−H]^−^;
								621[M−H−Ac−Ara(f)−GlcGlc]^−^
15	Rg_3_	C_42_H_72_O_13_	784.497	+	+	783.490	829.495	871[M+HCOO]^−^; 783[M−H−Ac]^−^; 621[M−H−Ac−Glc]^−^;
								459[M−H−Ac−GlcGlc]^−^; B_1β_161[Glc−H]^−^
16	Rg_2_	C_42_H_72_O_13_	784.497	+	-	783.490	829.495	783[M−H]^−^; 637[M−H−Rha]^−^; 475[M−H−Rha−Glc]^−^;
								391[M−H−Rha−Glc−C_6_H_12_]^−^
17	Rg_5_	C_42_H_70_O_12_	766.487	+	+	765.480	811.484	811[M+HCOO]^−^; 765[M−H]−; 603[M−H−Glc]^−^;
								441[M−H−Glc−Glc]^−^; ^1,3^A_2β_221; B_1β_161[Glc−H_2_O−H]^−^
18	Rg_6_	C_42_H_70_O_12_	766.487	+	+	765.480	811.484	811[M+HCOO]^−^; 765[M−H]^−^; 619[M−H−Rha]^−^;
								457M−H−Rha−Glc]^−^
19	Rk_1_	C_42_H_70_O_12_	766.487	+	+	765.4795	977.533	765.4755 [M−H]^−^; 603[M−H−Glc]^−^; 441[M−H−GlcGlc]^−^;
								^1,3^A_2β_221; B_1β_161[Glc−H_2_O−H]^−^
20	F4(Rg_4_)	C_42_H_70_O_12_	766.487	+	+	765.4795	811.484	765[M−H]^−^; 619[M−H−Rha]^−^; 457[M−H−Rha−Glc]^−^;
								^1,5^A_2β_279; ^1,3^A_2β_205; ^0,2^A_1β_101

Note: Glc: β-D-glucose, Ara (p): α-L-arabinose (pyranose), Ara (f): α-L-arabinose (furanose), Xyl: β-D-xylose, Rha: α-L-rhamnose, mal: malonyl; Trends “+” means a common component, “-” means no common component.

**Table 3:** Determination of soluble components in RG-CE and SGD-CE (*n* = 3).

No.	RT (min)	Compounds	Molecular Formula	Relative Contents (%)		CAS
				RG-CE	SGD-CE	
1	5.746	p-Xylene	C_8_H_10_	1.038	5.470	106-42-3
2	7.504	Octanal	C_8_H_16_O	0.121	-	124-13-0
3	7.782	Octane, 3,3-dimethyl-	C_10_H_22_	0.149	-	4110-44-5
4	8.358	Nonane, 4,5-dimethyl-	C_11_H_24_	0.327	1.016	17302-23-7
5	8.682	Ethane, hexachloro-	C_2_H_l6_	0.189	-	67-72-1
6	8.775	Oxirane,[[(2-ethylhexyl)oxy]methyl]-	C_11_H_22_O_2_	0.065	-	2461-15-6
7	8.95	4-Nonenal,(E)-	C_9_H_16_O	0.165	-	2277-16-9
8	9.102	Nonanal	C_9_H_18_O	0.265	0.949	124-19-6
9	9.238	2,3-Dimethyldecane	C_12_H_26_	0.091	-	17312-44-6
10	9.255	Octane, 2,3,6,7-tetramethyl	C_12_H_26_	-	0.430	52670-34-5
11	9.539	Benzenamine,2-ethyl-	C_8_H_11_N	0.115	-	578-54-1
12	10.148	3-(1,3,5-Cycloheptatrien-7-yl)-2,4-pentanedione	C_12_H_14_O_2_	0.259	-	65548-56-3
13	10.43	Naphthalene	C_10_H_8_	0.126	0.550	91-20-3
14	10.63	2-Methyltetracosane	C_25_H_52_	0.132	-	1560-78-7
15	10.642	Decanal	C_10_H_20_O	-	0.501	112-31-2
16	10.741	Undecane, 2,5-dimethyl	C_13_H_28_	0.155	-	17301-22-3
17	10.867	Benzaldehyde, 3,4-dimethyl	C_9_H_10_O	0.281	-	5973-71-7
18	11.181	Nonane, 3-methyl-5-propyl	C_13_H_28_	0.118	0.387	31081-18-2
19	11.343	Caprolactam	C_6_H_11_NO	1.150	6.122	105-60-2
20	11.466	Decane, 3,6-dimethyl-	C_12_H_26_	-	0.833	17312-53-7
21	11.701	Dodecane, 4,6-dimethyl	C_14_H_30_	0.504	-	61141-72-8
22	11.705	Pentadecane	C_15_H_32_	-	1.555	629-62-9
23	11.82	3-(Hydroxy-phenylmethyl)-2,3-dimethyloctan-4-one	C_17_H_26_O_2_	0.232	-	1000192-68-2
24	11.962	Tridecane	C_13_H_28_	0.090	-	629-50-5
25	12.081	Carbonic acid, decylundecyl ester	C_22_H_44_O_3_	0.145	-	1000383-16-0
26	12.465	Dodecane, 2,6,11-trimethyl	C_15_H_32_	0.092	-	31295-56-4
27	12.833	10-Methylnonadecane	C_20_H_42_	0.102	-	56862-62-5
28	13.313	Niacinamide	C_6_H_6_N_2_O	0.168	-	98-92-0
29	13.317	Tetradecane	C_14_H_30_	-	0.570	629-59-4
30	13.402	Vanillin	C_8_H_8_O_3_	0.486	-	121-33-5
31	13.999	Carbonic acid, decyltridecyl ester	C_24_H_48_O_3_	-	0.535	1000383-16-2
32	14.12	2,6,10-Trimethyltridecane	C_16_H_34_	0.250	-	3891-99-4
33	14.206	Dodecane, 2-methyl-	C_13_H_28_	0.126	-	1560-97-0
34	14.776	2,4-Di-tertbutylphenol	C_14_H_22_O	1.578	3.713	96-76-4
35	14.855	Butylated Hydroxytoluene	C_15_H_24_O	0.134	-	128-37-0
36	14.995	Tetradecane,4-methyl-	C_15_H_32_	-	0.393	25117-24-2
37	15.113	Hexadecane, 2,6,11,15-tetramethyl	C_20_H_42_	0.261	-	504-44-9
38	15.115	Heptadecane	C_17_H_36_	-	0.814	629-78-7
39	15.464	Ditetradecyl ether	C_28_H_58_O	0.097	-	5412-98-6
40	15.762	Butyrovanillone	C_11_H_14_O_3_	0.982	0.546	64142-23-0
41	15.828	Diethyl Phthalate	C_12_H_14_O_4_	0.170	-	84-66-2
42	17.19	Parbenate	C_11_H_15_NO_2_	-	1.000	10287-53-3
43	17.384	1,4-Azulenediol,1,2,3,3a,4,5,6,8aoctahydro-1,4-dimethyl-7-(1-methylethyl)-,(1R,4S)-	C_15_H_26_O_2_	0.958	-	2117730-73-9
44	17.464	(E)-4-(3-Hydroxyprop1-en-1-yl)-2-methoxyphenol	C_10_H_12_O_3_	1.252	-	32811-40-8
45	17.56	Tetradecane, 2,6,10-trimethyl	C_17_H_36_	-	1.039	14905-56-7
46	17.656	Panaxatriol	C_30_H_52_O_4_	0.600	-	32791-84-7
47	17.96	(4S,8aR,9R,12S,12aR)-9,12-Dihydroxy-4-methyldodecahydro-2Hbenzo[d]oxecin-2-one	C_14_H_24_O_4_	0.324	-	1010482-41-6
48	18.027	Octadecane	C_18_H_38_	-	0.907	593-45-3
49	18.119	Incensole oxide, methyl ether	C_21_H_36_O_3_	0.299	-	1000513-23-2
50	18.255	cis-Z-.alpha.-Bisabolene epoxide	C_15_H_24_O	3.897	-	1000131-71-2
51	18.513	Uvidin C	C_15_H_26_O_3_	0.313	-	74635-85-1
52	18.642	6-(2-Hydroxypropan-2-yl)-4,8a-dimethyl2,3,4,6,7,8-hexahydro1H-naphthalen-1-ol	C_15_H_26_O_2_	0.370	-	2061568-37-2
53	18.705	E-8-Methyl-9- tetradecen-1-ol acetate	C_17_H_32_O_2_	0.249	-	1000130-81-4
54	18.838	2,4,7,14-Tetramethyl4-vinyltricyclo[5.4.3.0(1,8)] tetradecan-6-ol	C_20_H_34_O	0.337	-	1000193-31-2
55	19.06	Longifolenaldehyde	C_15_H_24_O	0.722	-	19890-84-7
56	19.209	3,6-Dimethoxy1a,2,2a,3,6,6a,7,7aoctahydro-1-oxacyclopropa[b]naphth alene	C_12_H_18_O_3_	1.416	1.118	1010191-51-4
57	19.278	2,4-Dioxohexahydro1,3,5-triazine	C_3_H_5_N_3_O_2_	4.861	-	1000484-54-9
58	19.397	7,9-Di-tert-butyl-1-oxaspiro(4,5)deca-6,9-diene-2,8-dione	C_17_H_24_O_3_	0.394	1.472	82304-66-3
59	19.513	Pyrrolo(1,2- a)pyrazine-1,4-dione, hexahydro-3-(2-methylpropyl)-	C_11_H_18_N_2_O_2_	0.712	-	5654-86-4
60	19.669	n-Hexadecanoic acid	C_16_H_32_O_2_	1.429	-	21096
61	20.443	(S,Z)-Heptadeca-1,9- dien-4,6-diyn-3-ol	C_17_H_24_O	7.028	-	81203-57-8
62	20.881	9-Octadecenenitrile	C_18_H_33_N	1.012	-	112-91-4
63	20.997	Heneicosane	C_21_H_44_	-	5.036	629-94-7
64	21.563	Octadecanoic acid	C_18_H_36_O_2_	0.377	-	21128
65	21.798	Panaxydol	C_17_H_24_O_2_	11.258	-	72800-72-7
66	21.993	cis-13-Eicosenoic acid	C_20_H_38_O_2_	0.515	-	17735-94-3
67	22.248	1,3,5-Cycloheptatriene,7,7-dimethyl-2,4-diphenyl	C_21_H_20_	0.518	-	1000156-99-8
68	22.665	Benzene,1,1’,1’’,1’’’-(1,2,3,4-cyclobutanetetrayl)tet rakis	C_28_H_24_	7.267	-	806-90-6
69	22.771	Octadecane, 3-ethyl-5-(2-ethylbutyl)-	C_26_H_54_	-	9.909	55282-12-7
70	23.486	Benzene, 1,1’,1’’-(1-propanyl-3-ylidene)tris	C_21_H_20_	8.459	-	19120-39-9
71	23.553	1,9-Diphenyl-1,3,5,7-nonatetraene	C_21_H_20_	9.967	9.320	1010326-78-2
72	23.774	14-Deoxy-11,14-didehydroandrographolide	C_20_H_28_O_4_	1.717	-	42895-58-9
73	23.91	Phenol, 2,2’-methylenebis[6-(1,1-dimethylethyl)-4-methyl	C_23_H_32_O_2_	5.375	14.025	119-47-1
74	24.41	Pentacosane	C_25_H_52_	-	11.245	629-99-2
75	24.415	(Z)-Docos-9-enenitrile	C_22_H_41_N	1.719	-	1000465-48-0
76	24.533	Hexadecanoic acid, 1-(hydroxymethyl)-1,2-ethanediyl ester	C_35_H_68_O_5_	-	9.450	761-35-3
77	24.543	Hexadecanoic acid, 2-hydroxy-1-(hydroxymethyl)ethyl ester	C_19_H_38_O_4_	2.860	-	23470-00-0
78	25.185	Nonacosane	C_29_H_60_	-	11.097	630-03-5
79	26.241	Octadecanoic acid, 2,3-dihydroxypropyl ester	C_21_H_42_O_4_	1.085	-	123-94-4
80	26.874	13-Docosenamide,(Z)-	C_22_H_43_NO	0.836	-	112-84-5
81	27.407	Squalene	C_30_H_50_	0.962	-	111-02-4
82	28.36	Oxirane, 2,2’-[(1-methylethylidene)bis(4,1-phenyleneoxymethylene)]bis	C_21_H_24_O_4_	0.584	-	1675-54-3

## Data Availability

The datasets used and/or analyzed during the present study are available from the corresponding author upon reasonable request.

## Supplementary Materials

The following supporting information can be downloaded at https://www.mdpi.com/article/10.3390/molecules27238202/s1: Figure S1, plots of enzyme activity. The enzyme activity indicates the change in absorbance at 475 nm at different concentrations of RG and SGD. The final L-DOPA concentration was 1 mg/mL. (A1 RG-CE; A2 RG-EAE; B1 SGD-FDP; B2 SGD-WE; B3 SGD-NBE; B4 SGD-EAE; B5 SGD-CE). Figure S2, Lineweaver–Burk plot. The different concentrations at RG and SGD are shown. (A1 RG-CE; A2 RG-EAE; B1 SGD-FDP; B2 SGD-WE; B3 SGD-NBE; B4 SGD-EAE; B5 SGD-CE). Figure S3, plots of enzyme activity. The enzyme activity indicates the change in absorbance at 410 nm at different concentrations of RG-NBE and SGD-NBE. (A RG-NBE; B SGD-NBE). Figure S4, Lineweaver-Burk plot in different concentrations at RG-NBE and SGD-NBE. (A RG-NBE; B SGD-NBE). Figure S5, total ion chromatography diagrams of NBE components from RG and SGD. (A RG-NBE; B SGD-NBE).
