# Supplementary material for: By-Product of the Red Ginseng Manufacturing Process as Potential Material for Use as Cosmetics: Chemical Profiling and In Vitro Antioxidant and Whitening Activities
Source: Molecules. 2022 Nov 24;27(23):8202. doi: 10.3390/molecules27238202 (PMC9736987; doi:10.3390/molecules27238202)
Supplement: Supplementary file 1 [file molecules-27-08202-s001.zip › molecules-2039818-supplementary.pdf]

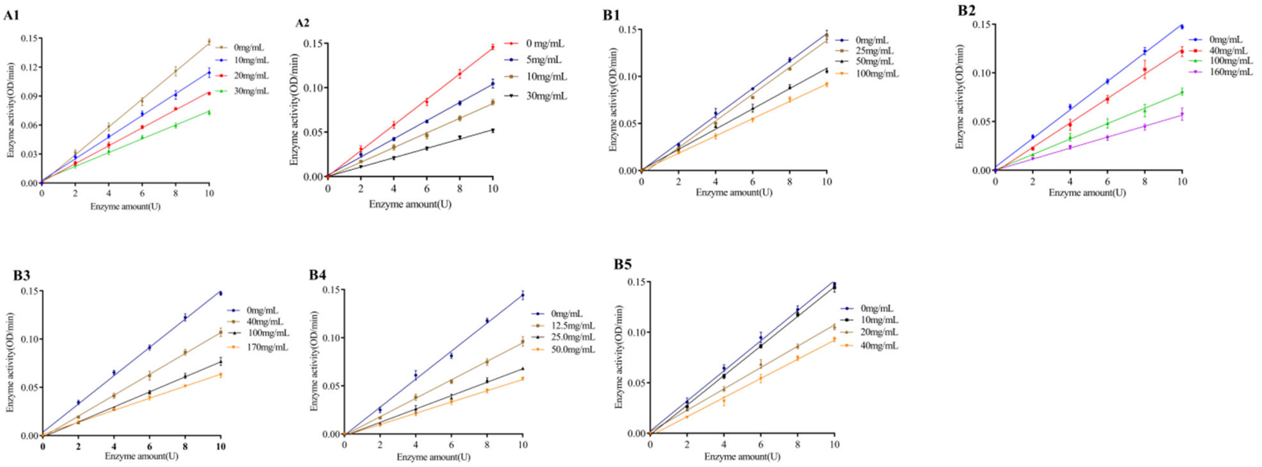

Figure S1. Plots of enzyme activity. The enzyme activity indicates the change in absorbance at 475 nm at different concentrations of RG and SGD. The final L-DOPA concentration was 1 mg/mL. (**A1** RG-CE; **A2** RG-EAE; **B1** SGD-FDP; **B2** SGD-WE; **B3** SGD-NBE; **B4** SGD-EAE; **B5** SGD-CE)

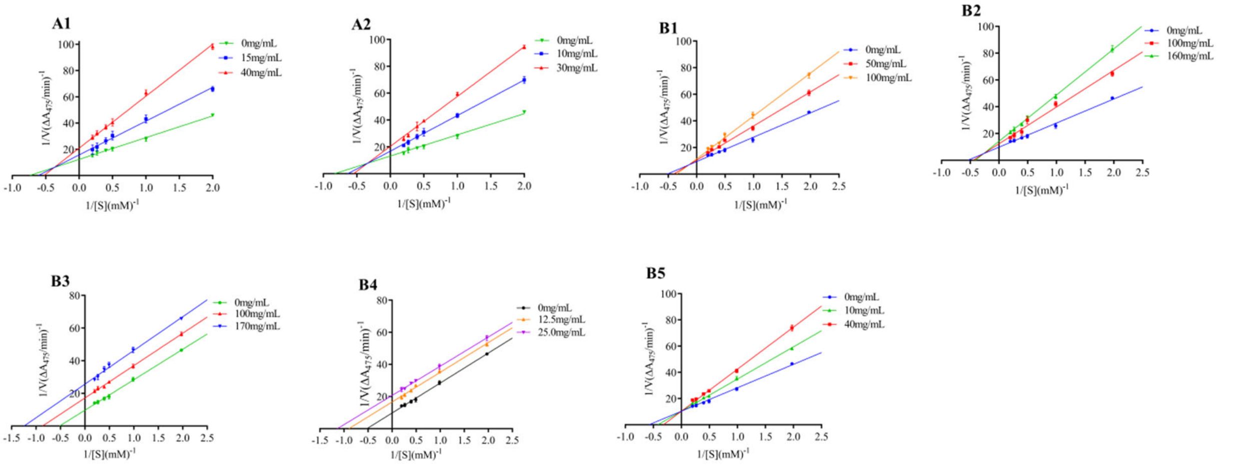

Figure S2. Lineweaver-Burk plot. The different concentrations at RG and SGD. (**A1** RG-CE; **A2** RG-EAE; **B1** SGD-FDP; **B2** SGD-WE; **B3** SGD-NBE; **B4** SGD-EAE; **B5** SGD-CE)

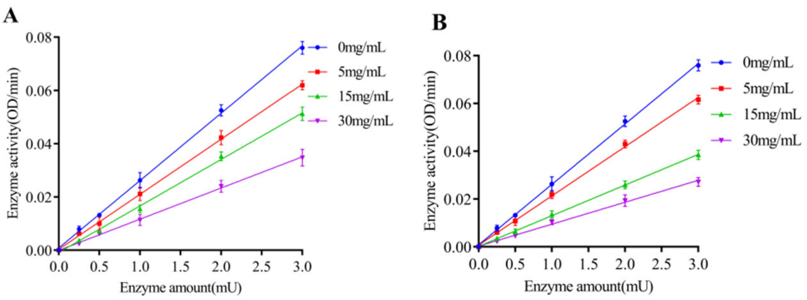

Figure S3. Plots of enzyme activity. The enzyme activity indicates the change in absorbance at 410

nm at different concentrations of RG-NBE and SGD-NBE. (**A** RG-NBE **B** SGD-NBE)

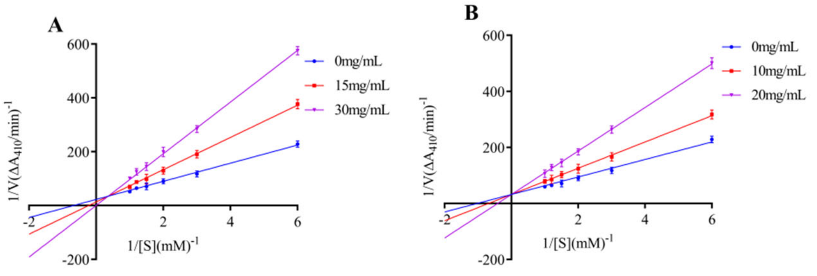

Figure S4. Lineweaver-burk plot in different concentrations at RG-NBE and SGD-NBE. (**A** RG-NBE; **B** SGD-NBE)

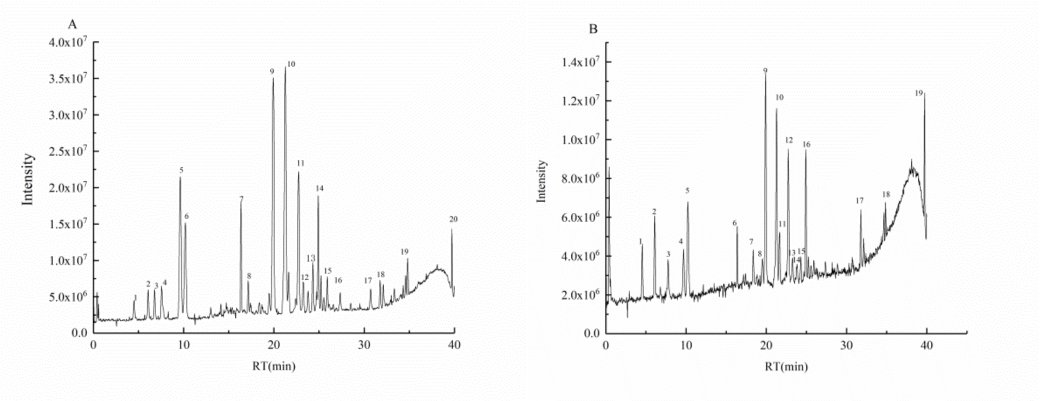

Figure S5. Total ion chromatographydiagrams of NBE components from RG and SGD. (**A** RG-NBE; **B** SGD-NBE)
